# Influence of climate change and accidents on perception differs among energy technologies

**DOI:** 10.1093/pnasnexus/pgaf079

**Published:** 2025-03-07

**Authors:** Guillaume F L’Her, Nickolas A Duncan, Hank C Jenkins-Smith, Mark R Deinert

**Affiliations:** Nuclear Science and Engineering, The Colorado School of Mines, Golden, CO 80401, USA; Nuclear Science and Engineering Research Center, United States Military Academy, West Point, NY 10996, USA; Institute for Public Policy Research and Analysis, University of Oklahoma, Norman, OK 73019, USA; Nuclear Science and Engineering, The Colorado School of Mines, Golden, CO 80401, USA; Payne Institute for Public Policy, The Colorado School of Mines, Golden, CO 80401, USA

## Abstract

Risk perceptions of energy systems, and their evolution under climate change and after accidents, affect public acceptance of generation technologies. Despite this, little is understood about how such factors impact public perception at different timescales and the drivers for perception. We use state-of-the-art natural language processing to measure temporal changes in sentiment toward energy technologies using the full Twitter archive for 2009–2022. We find that perception of natural gas and wind has changed little as discussion of climate change on social media increased. However, climate-linked sentiment toward coal, solar, and hydropower has become more negative, while that for nuclear has improved. We also find that all generation technologies experience a drop in supportive discourse after definable accidents, but this typically rebounds with a half-life of <3 days. Yet, nuclear power is an exception in how it reacts to large-scale events. After Fukushima, sentiment returned to its positive preaccident levels with an 11.3-month relaxation half-life.

**Significance Statement**

Public perception of energy technologies is crucial for their acceptance and development, particularly in the context of climate change and postaccident scenarios. This study employs advanced natural language processing techniques to analyze Twitter discourse from 2009 to 2022, revealing how sentiments toward different energy sources evolve over time. While perceptions of natural gas and wind remain relatively stable, coal, solar, and hydropower are increasingly viewed negatively in climate-related discussions. Notably, sentiment toward nuclear power improved over the period studied, despite a significant drop following the Fukushima disaster. This research highlights the dynamic nature of public sentiment toward energy technologies, providing insights into the factors driving these changes and informing future communication strategies.

## Introduction

Risk perception is an essential part of the survival mechanisms of any living species (1). However, while researchers focus on quantifying risks objectively, most people use an intuitive approach (2) which is based on past experiences and the lessons learned (1). As a result, people often have difficulty putting risks in context, and this is especially true for rare events or ones with delayed consequences and notably for complex infrastructure systems on which modern society rests.

In the second half of the twentieth century, nuclear war was the existential threat at the forefront of public fears and this also contributed to a disproportionate risk perception for radioactivity relative to other things (3, 4). This in turn strongly impacted people's perception and disposition to low-carbon nuclear power, slowing the industry to a crawl in most regions of the world, and eventually causing a significant loss of construction know-how in developed countries (5). While fear of nuclear war and technology is still present today (6, 7), another existential threat has now taken a much larger role in the public place—climate change (8). Public perception of its current and future risks has been the driver for a global push to transition away from fossil fuels (9, 10). However, the adoption of energy technologies is affected not only by their attributes and economics, but also by political considerations (11, 12) where public acceptance can play a large role (13). In this context, risk perception and related discussions are particularly important and are affected not only by public concerns about the technology itself but also how people perceive its potential to address significant issues such as climate change. Some studies have explored public perceptions of low-carbon energy (14) and geoengineering technologies (15). However, these studies do not look at the drivers for perception or how perception changes over different timescales and in relation to increasing concern over climate change or to events such as accidents.

Conventional surveys have been widely used to measure the perception of risk related to energies in a population (16–18). Unfortunately, this approach cannot easily measure the dynamics of public perception in response to short timescale events such as accidents, and while some projects have shown important results (19), longitudinal studies performed to gauge long-term trends (20) can be very expensive, especially over diverse geographical regions. However, social media data can be used to understand public perception over both of these timescales (21, 22). Multiple studies have used Twitter data (and other social network data) to assess public sentiment toward various topics using unprompted data mining. This information can be used to monitor ongoing or past events, such as hurricanes, earthquakes, floods, epidemics, and industrial disasters (23–26). Social discourse network analysis using social media has also been performed to understand public narratives about nuclear power and who in these networks is supportive or against the technology (27). Recent advances in sentiment and text analysis using natural language processing allow for standardized quantification of emotional states and opinions from text (28), an approach validated against traditional methods such as the Gallup–Sharecare Well-Being Index survey (24, 25). While platforms such as Twitter may not yet capture the entire spectrum of sociodemographic space (29, 30), this is shifting (23). Some authors have noted that social media allows for a more “nuanced” views of energy technologies than do conventional survey methods (21).

While many studies have been conducted to investigate the perception of different energy technologies, no study has yet looked holistically at the time-dependent impact of climate change discussions and adverse events, how this varies between technologies, or the timescale of the resulting information cascades. Here, we use natural language processing and Twitter data to do exactly this. We quantify the impacts of climate change considerations on risk perception and the half-life of multiple information cascades of varying scales across different energy technologies. Tweets were filtered according to mainstream energy type (coal, nuclear, hydro, wind, and solar) using a list of keywords. All the original tweets from January 2009 and July 2022 containing at least one of the keywords were considered in this study, representing more than 26 million tweets from ∼4 million unique users. For each tweet, perception was assessed as positive or negative based on fine-tuning of a transformer model (RoBERTa) (31) with training data manually classified by three independent persons. A tweet was classified as positive if it showed the energy technology in a positive light, whether factual or subjective, and negative otherwise. All tweets were aggregated at relevant timescales (daily and monthly), and the proportion of positive tweets was computed at each time step. The evolution of positive attitudes toward an energy technology after a perturbation was measured using an exponential function. The half-life was defined as the time necessary to recover half of the positivity between a reference value and the most negative discourse point.

## Results

### Evolution of public discourse

**Fig. 1.**
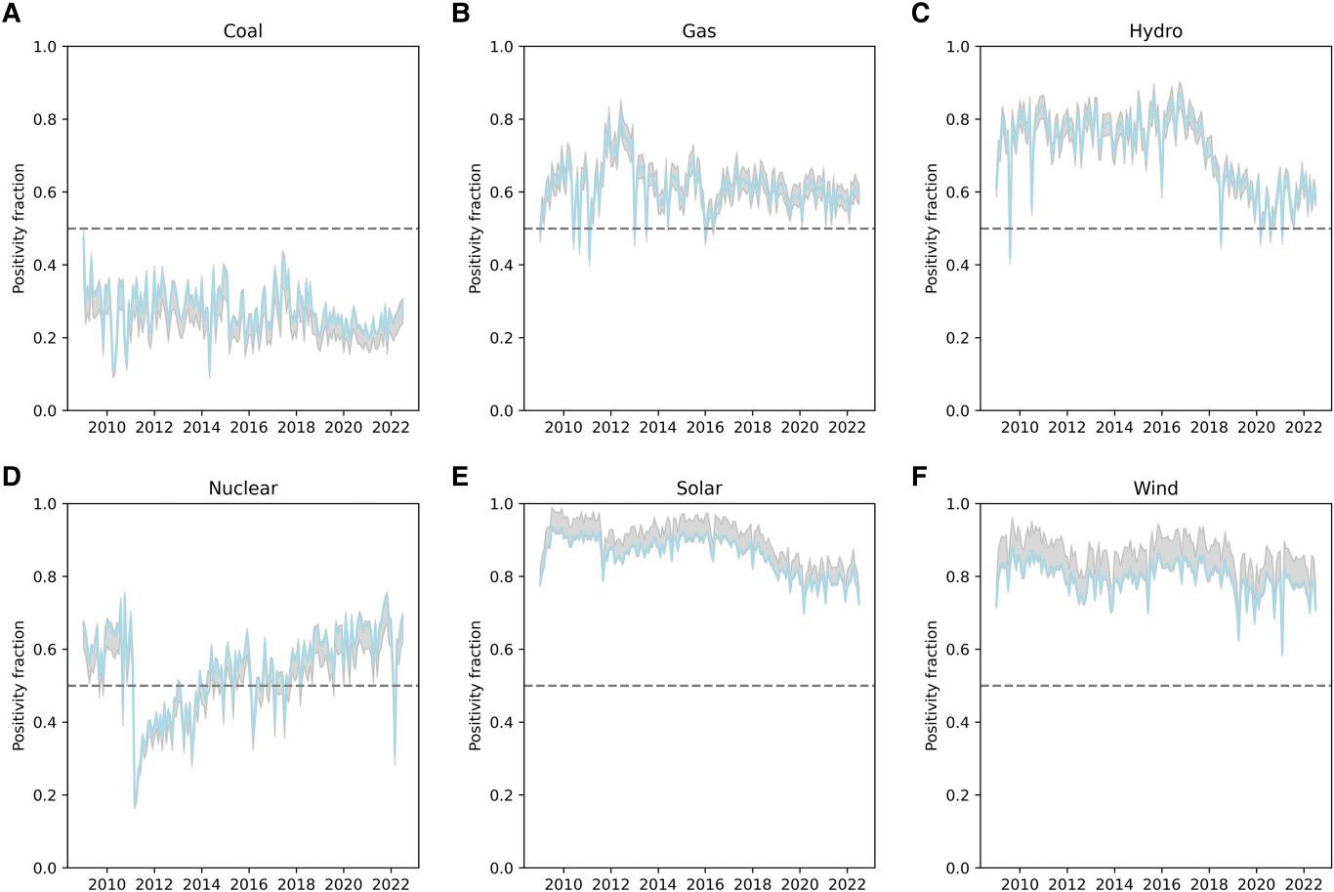
Public discourse support of mainstream energy technologies over time. The tweets are aggregated monthly, and the positivity fraction is plotted over time for each technology (A: coal, B: Natural gas, C: Hydroelectric, D: Nuclear, E: Solar, F: Wind). A score of 0 implies 0% of positive tweets about a given energy, and a score of 1 translates to 100% positive tweets during a given month. The gray dashed lines at 50% represent as many positive tweets as negative ones. The graphs cover the period January 2009 to July 2022. The shaded areas represent the model uncertainty based on the false-positive and false-negative rates observed during training, as indicated by the Matthews correlation coefficient measure.

Figure 1 shows the monthly support level seen in the public discourse from 2009 to summer 2022 for each of the six energy technologies considered in this study. Levels above 0.5 indicate positive perception (more than 50% of tweets are positive), and levels below 0.5 indicate negative perception (more than 50% of tweets are negative). In the last decade, public opinion toward coal, already negative, has degraded even more. Natural gas shows a stable supporting trend in the public online conversation. The data show that while discourse is not too “enthusiastic,” in contrast to solar and wind, exchanges on social media still present this technology in a positive light (hovering around 60% positive). The impact of rising prices, and potential disruptions to European natural gas supplies due to the Russia–Ukraine war, does not appear to change the narrative. “New renewable” energies, solar and wind, are seen in the most positive light, driven by encouraging opinions and a large relative growth over the decade. A drop in public support has nonetheless been observed on social media in the past couple of years. Hydroelectric power, a long-time staple of renewable energy, has exhibited an interesting fall in observed sentiment levels since 2016.

The data in Fig. 1 show tweets sentiment over time. While these represent the online discourse that users were exposed to, in some cases these data could be dominated by a smaller fraction of active users and mask the true sentiment levels of the online population. To isolate the latter, we looked at the average positivity exhibited by account holders (that tweeted about at least one of the energy technologies). The detail of this analysis is presented in Supplementary Information Note S1. We observe that a higher number of unique users tweet about nuclear energy than any other technology, hinting at a higher resonance of some energies due to perceived risks and concerns about being personally impacted. The user-specific results confirm the trend seen in the tweets-level study. While Table S1 combines all users over the entire analysis period, Table S2 shows the individual opinions in 2010 and Table S3 shows them in 2020. These two latter tables notably exhibit a fall in sentiment for hydroelectric power (a loss of 20% points) and show a more circumspect discourse on new renewables over time. The data also show the rebound of the nuclear industry after the Fukushima accident, with a similar share of Twitter users shining a positive light on nuclear energy in 2020 compared with 2010.

Of the technologies for which time-series sentiment was measured, nuclear power shows the largest drops, and these correspond to the Fukushima Daiichi accident and the takeover of the Zaporizhzhia nuclear power facility in March of 2022, as shown in Fig. 1D. As measured using tweets, sentiment toward nuclear power returned to pre-Fukushima levels and became even slightly more positive than hydroelectric power by 2019 and on par with natural gas. This long-term trend was dampened recently by the ongoing war in Ukraine, in which nuclear power plants (Chernobyl and Zaporizhzhia) were taken over. These events do not seem to have perturbed the ongoing narrative about nuclear energy on social media, as the positive sentiment rebounded back very quickly. It does, however, show that nuclear energy is extremely susceptible to negative news, which may increase the uncertainty with the long-term prospects for this technology in the eyes of private investors and political campaigns. The daily variability in the average public perception on social media displays more volatile opinions for nuclear and hydroelectricity compared with other energies, as given in Table S4. Coal and natural gas are slightly more stable, and new renewables show the lowest levels of day-to-day variability. This indicates how people online typically react to the perception of risks. Natural gas being the main competitor of nuclear energy for baseload power, this stability, along with a similarly positive view, is likely to impact the energy transition. However, the analysis also reveals a convergence of the daily variability over time for all energies, as shown in Fig. S8. This shows how opinions on Twitter have self-organized into a set of dominating talking points and removed nuance.

### Sensitivity of public discourse to adverse events

**Fig. 2.**
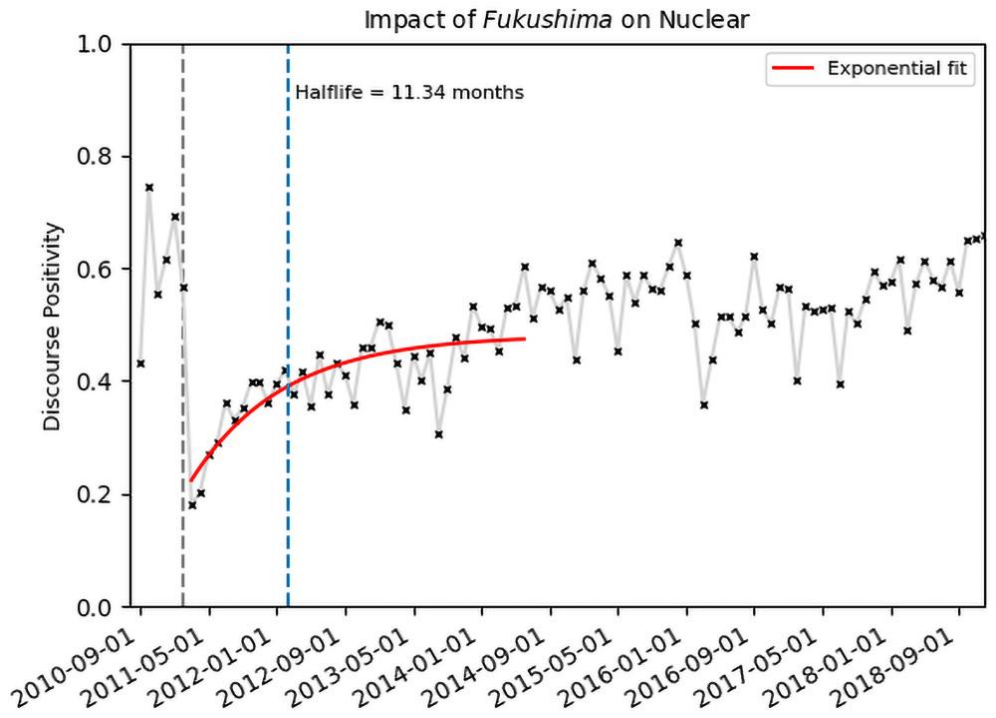
Monthly evolution of the Fukushima Daiichi accident on public discourse. The half-life (vertical dashed line) is defined as the amount of time before public discourse is back at 50% of initial levels after a perturbation, based on an exponential fit. The first vertical dashed line represents the month prior to the event for reference, February 2011 here.

To go beyond a daily variability evaluation and better assess risk perception toward the technology itself, we also measure the susceptibility of each energy to adverse events (incidents that made it to the media, Figs. S1–S7 in Supplementary Information Note S2) and assess the impact duration of such events on how each energy type is perceived on social media. The online public response to energy-related events depends on the technology being discussed (e.g. nuclear, wind, etc.). To quantify this, we define and compute a “half-life” for the sentiment recovery time after adverse events. This is defined as the time needed to recover half of the dip between the average *support* right before the perturbation and the most negative data point (e.g. Fig. 2). We identify multiple events for each technology (examples shown in Supplementary Information Note S2) and compute their respective recovery time in the online public discourse by calculating their half-life. Catastrophic and deadly events related to coal or natural gas often do not register on the public discourse positivity, as given in Table 1. This is also seen in hydroelectricity-related events, only 31% of those impacting the discourse. Nuclear energy is more sensitive to small incidents, with 50% of the identified events registering on social media. All these technologies exhibit similar perturbation half-lives for small-scale events when they impact online discourse. While online sentiment about solar and wind is resilient to maintenance or installation accidents, some rare events can still significantly impact public discourse. The Texas winter storm in February 2019 and the Ivanpah concentrated power plant fire are two examples of these.

**Table 1.** Mean half-life of events that impacted online public discourse per technology.

Technology	Event registering (%)	Mean half-life (days)
Coal	35.9	1.54 ± 1.09
Natural gas	25.0	1.36 ± 1.17
Nuclear	50.0	1.23 ± 0.97
Hydropower	31.0	1.48 ± 0.69

The events were identified by using the results from the first 15 pages of results from Google News for each year for given keywords (Supplementary Information Note S2). The half-life SDs are given using the population SD.

In addition to a greater sensitivity to small inconsequential incidents, nuclear energy also demonstrates a severe weakness to the long-term impacts of a large single event. Other industries do not exhibit this long-lasting impact behavior based on public concern of personal health consequences. Support toward nuclear energy dropped significantly after the Fukushima Daiichi accident in March 2011 which followed the Tohoku earthquake and tsunami, as shown in Fig. 2. We calculate that Fukushima created a perturbation on the nuclear energy discourse with a half-life of 11.3 months and a total recovery time >3 years, as shown in Fig. 2. This suggests that the real, quantifiable consequence of a nuclear accident is not the principal driver of public opinion (as those abated quickly with Fukushima). However, sentiment levels for nuclear power did rebound. This is consistent with the findings of historical surveys which showed that changes in public perception were temporary after major accidents (32). The rapid drop and rebound in Twitter sentiment toward nuclear power that followed the Russian seizing of the Chernobyl and Zaporizhzhia Nuclear Power Stations in Ukraine in 2022, as shown in Fig. 1, showed that the effect on social media was transient. This difference in half-life might result from a difference in perception when there is no release of radioactivity. This points to the need to avoid hasty decisions based on immediate public perceptions as it relates to long-term energy projects, as was, for example, the case of the German energy strategy.

### Climate change as a driver of risk perception

**Fig. 3.**
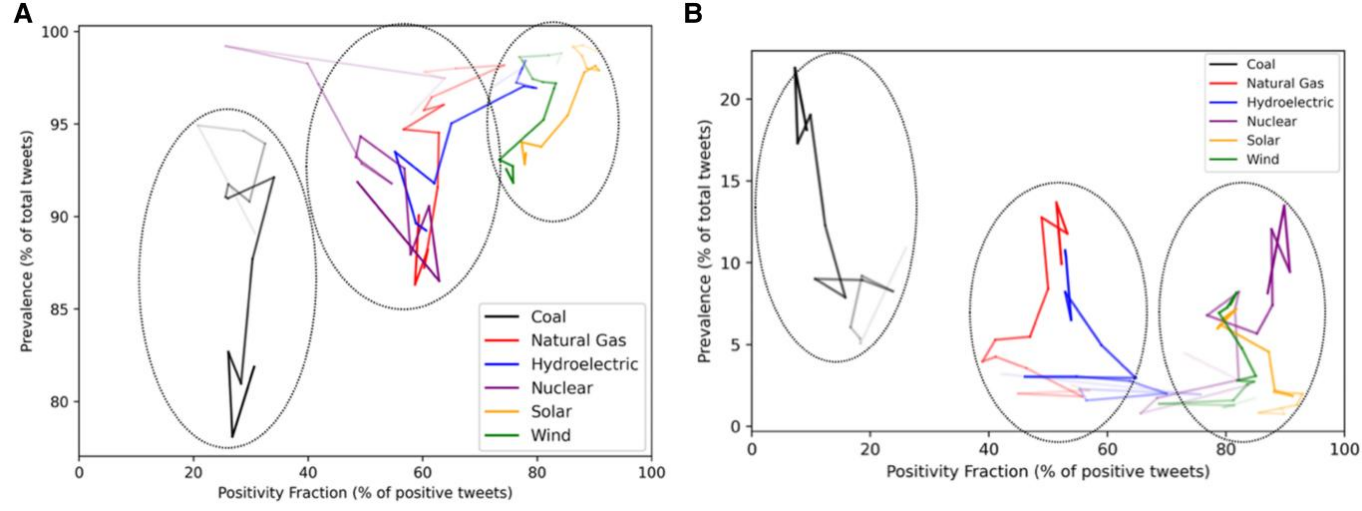
Time walks of positivity and prevalence of energy discussions for (A) nonclimate-related topics and (B) climate-related topics, 2009–2022. Each direction change represents the data aggregated for a given year. Time evolution is shown using fading transparency. The clusters shown with the dotted regions are based on a *K*-mean clustering analysis (Fig. S8 in Supplementary Information Note S3), with a silhouette score of 0.69 for the climate-related topics and 0.65 for the nonclimate-related topics and statistical difference validated by an ANOVA test (Supplementary Information Note S3).

The perception of climate change impacts and necessary mitigation is an important large-scale risk driver that sits behind the need for energy transition and associated public discussions. Figure 3 shows how the discourse related to energies and climate topics evolved over time on social media. Over time, the data show that climate is an ever more prevalent facet of discussion for all energy technologies. While in 2009 climate was seldom explicitly mentioned for most energies, with a slight exception for coal, this had changed by the end of the following decade. Perhaps, counterintuitively, hydroelectricity can be seen in a “neutral” group along with natural gas, driven by environmental impact concerns as well as the methane gas emissions issue. When explicitly excluding climate topics from the tweet population (Fig. 3, left), three distinct groups of energies emerge: negative (coal), neutral (natural gas, nuclear, and hydroelectricity), and positive (solar and wind) supportive perception. However, considering these climate-related tweets (Fig. 3, right) shows nuclear energy joining wind and solar in the “positive” group and even overtaking their positivity levels in recent years. Only nuclear energy exhibits such a switch in its positivity group based on climate considerations.

**Fig. 4.**
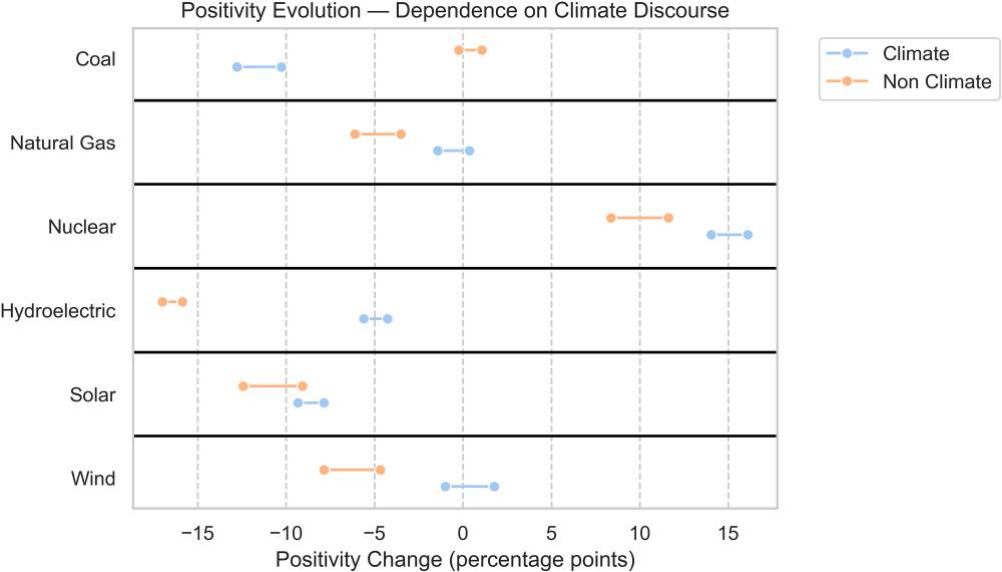
Evolution of energy technology support when climate topics are mentioned over 2009–2022. The ranges shown represent the range of opinion change as we consider variable aggregated yearly periods to calculate the temporal evolution (three years: 2009–2012 vs 2019–2022; four years: 2009–2013 vs 2018–2022; and five years: 2009–2014 vs 2017–2022). The Hodges–Lehmann estimator was derived for the computed temporal evolutions, showing statistically significant differences between the climate- and nonclimate-related positivity evolution points for all energy technologies (Table S4 in Supplementary Information Note S4).

Over the last decade, the online opinion on nuclear energy has shifted significantly, coming back from the Fukushima accident negative perception, and now sits in the same category as new renewables energies such as solar and wind when it comes to climate change. Figure 4 shows the average temporal evolution of supportive perception for climate vs nonclimate public discourse for each energy technology. As could be expected, coal energy exhibits a strong prevalence and negativity to climate topics, a main driver behind the negative support evolution for this technology. Driven in part by the correction from the half-life of the Fukushima accident at the start of last decade, nuclear energy presents a positive evolution of support for nonclimate-related topics. The increase in support toward nuclear energy is even more visible when considering climate-related topics. Only nuclear energy exhibits a consistent increase in positivity over time in tweets where climate change is discussed. In these social media discussions, coal, hydroelectricity, and remarkably solar energy display a negative evolution of perception, whereas natural gas and wind energy show relatively constant support. Although climate considerations are not as negative as nonclimate topics for these energies, it is insufficient to drive their positive perception further upward on social media. As it stands, climate discourse only prevents the opinions toward these energies from falling even faster. This demonstrates a significant shift in public opinion that could have strong effects on energy transition policies. The risks presented by climate change resonate more and more with the public, and this translates to support for nuclear power. This has been ongoing for the last few years and growing strongly with climate change concerns. Our results show this trend to be likely to continue, as long as the nuclear industry can work on better risks communication and plant safety.

The shift in perception of nuclear energy is mirrored in broader public opinion research. Dehner et al. (33) found that nuclear energy is perceived as part of the solution to climate change, with those more concerned about climate change exhibiting higher support for nuclear energy. Similarly, Bohdanowicz et al. (34) demonstrated how knowledge about climate change and nuclear energy positively correlates with support, particularly in Poland. Corner et al. (35) noted the role of conditional acceptance, where concern over climate change and energy security has led to increased, albeit reluctant, support for nuclear power as a low-carbon solution.

There are several limitations with the current study. Online social media, and more explicitly Twitter, does not yet represent the actual population demographics. Nontech-savvy people are inherently left out (36). Only English tweets are considered and classified here, and opinions given in other languages, reflecting dispositions in other countries, are ignored. Conversely, this implies that this study expands the understanding of risk perception of energy technologies in light of climate concerns beyond the United States, potentially limiting country-specific policy implications. The algorithms used for classifying tweets in this study are not well suited to understand certain nuances of language that can change the underlying meaning. A sarcastic tweet about a technology can be classified as positive or negative even when the opposite sentiment was intended by the user. Only Twitter data are used here. While usage is significant, it has been dropping over the past decade, with the average age of accounts increasing. This aging trend follows the decrease in sentiment SDs shown in Fig. S9 (Supplementary Information Note S5), suggesting perhaps a decrease in opinion diversity over time. Additionally, a potential change in demographic spectrum on the platform (following the acquisition of Twitter in April 2022) may also affect detected trends over time. News media were used to identify events with which a sentiment half-life could be measured. However, this is certainly only a subset of all events and their appearance in the media would suggest they were large enough to warrant media attention. Access to Twitter data for research purposes has fluctuated over time but became quite restricted in 2023. If this persists, other sources of social media data will be needed to help provide insights into public perception of energy technologies and climate. The tweets in this study were coded as either negative or positive. However, one could also envision a neutral category into which purely factual tweets could be categorized. This could help to eliminate coding errors (positive or negative) associated with factual statements where accurately assessing the tweets would potentially require knowledge of previous postings by a user, which was not encompassed by the algorithms used here.

## Conclusion

Risk perception is a strong driver of public acceptance and support for energy technologies. In the public eye, risk can include accidental events related to specific technologies, as well as global scale existential threats such as climate change. Natural language processing and social media can be used to understand temporal variations in how energy technologies are perceived, how perception recovers after adverse events, and how it changes when world-altering climatic crises are considered. We show that the online discussion of nuclear energy largely recovered from the Fukushima accident within 3 years. We also show that views on hydroelectric power have fallen since 2016 and that wind and solar are the most positively perceived technologies overall.

All energy technologies are seen to be susceptible to at least some adverse events, with a short-term event half-life consistent across technology, up to 3 days. The number and scale of the events that registered varied strongly by energy type, with coal showing great resilience to even catastrophic events while nuclear power showed sharp reactions to even small ones. Nuclear is the only technology showing long-term perturbations to sentiment with a recovery half-life of 11.3 months to the Fukushima accident. The events in Ukraine following the Russian invasion caused a significant (although transient) drop in sentiment for nuclear power, underscoring the vulnerability of the nuclear industry to adverse events and its higher inherent risk perception.

A topic modeling analysis revealed that nuclear energy has joined new renewables to fight climate change in the public eye, especially since 2017. More importantly, as climate discussions become more prevalent online, we demonstrate that only nuclear energy shows a correlated increase in positive perception. Other energies exhibit either a constant (natural gas, wind) or even a negative (coal, hydroelectricity, and solar) correlation between perception and climate considerations. This is an important finding that indicates an ongoing public switch from accepting natural gas as a safe alternative for baseload power and believing in the new renewables’ potential, to preferring the development nuclear energy in the face of existential risks from climate change issues. Whether nuclear energy will be able to capitalize on this early signal of growing public support on social media and mitigate the heightened sensitivity to adverse event remains to be seen.

## Methods

The Twitter Academic Application Program Interface (API) allows authorized users to query the entirety of the Twitter data, whether relative to users of the platform or their posts (tweets). The monthly 10 million tweets quota and upstream filtering capabilities offered by the Twitter Academic API were used to pull all the relevant tweets, over the course of several months. Twitter allows users to post original tweets, or to share an existing tweet (retweet). Due to the limited rate of download, only the original tweets were obtained, and the retweets and quotes were ignored. However, each original tweet carries some metrics information such as the number of times it was retweeted or liked. This can be used to expand the dataset, although not without some loss of information (location, time, and user).

More than 26 million original tweets, posted by around 4 million unique users, are downloaded across six technologies: coal, natural gas, hydroelectricity, nuclear, wind, and solar. The tweets cover the time period January 2009 to July 2022. Table S5 in Supplementary Information Note S6 displays the keywords used to filter the tweets for each energy. The tweets are then cleaned to remove all emoticons and URLs. Some noisy tweets were additionally identified and removed (e.g. home weather station automatic posts, astronomy-related solar system topics, etc.).

To measure the positivity of social media public discourse for each energy technology, the data were classified into *support* and *oppose*. The *support* category is defined as a positive answer to the question: *Is the tweet shining a positive light, factual or subjective, on the considered energy?*, and the *oppose* category represents the complement. Tables S6–S11 in Supplementary Information Note S7 show examples of this classification for the different energies. For each energy technology, a few known partisan hashtags (e.g. #nuclear4climate for Nuclear *support* or #countoncoal for Coal *support*, etc.) and prominent accounts with known views (e.g. Greenpeace, Coal Coalition, etc.) are identified, and their data pulled to form automatic training data. This training dataset is completed by randomly sampled and manually classified tweets. Three people independently classified the randomly sampled tweets in order to mitigate inherent bias. Only the tweets categorized in agreement between every human classifier were used in the training sets. After eliminating the manually classified tweets that were not in complete agreement, more than 12,000 tweets remain, close to uniformly distributed across the six mainstream energy technologies of interest. A separate training set is consequently obtained for each energy technology. In each case, 70% of the labeled data are used to form the training set, 15% to build a validation set, and the remaining 15% to generate an unseen testing dataset. The training sets are then augmented to balance the ratio of *support/oppose* labels. This augmentation is performed using the NLPAug library (37), using a substitution method based on the fast DistilBERT transformer architecture (38).

The six models (one for each energy technology) are then trained on a Tesla V100 GPU, by fine-tuning the RoBERTa transformer (Fig S10 in Supplementary Information Note S8). Table 2 shows the size of the training set used for each technology, after balancing augmentation, and the model performance, evaluated using the Matthews correlation coefficient (39), ideal for binary classification problems. The presented models may be improved upon, notably for coal energy classification. However, they can all be deemed to be quite efficient at correctly predicting the support category prevalence for a large-scale study as performed in this study. The model uncertainties are computed using the false-positive and false-negative rates observed during training, as indicated by the Matthews correlation coefficient measure. As such, the CI of the model response for each energy technology fine-tuning is given by $\left[N_{\mathrm{pos}}\left(1-\frac{\;f_{p}}{t_{p}+f_{p}}\right),\;N_{\mathrm{pos}}\left(1+\frac{\;f_{n}}{t_{n}+f_{n}}\right)\right]$, where $f_{p},\;f_{n},\;t_{p},\;\;\mathrm{and}\;t_{n}$ are the false-positive, false-negative, true-positive, and true-negative results, respectively, observed during training.

**Table 2.** Machine learning model performance.

Model	Number of training tweets (support; oppose)	Matthews correlation coefficient
Coal	(3,918; 4,074)	0.74
Natural gas	(2,743; 2,245)	0.89
Nuclear	(9,012; 10,731)	0.90
Hydropower	(7,587; 5,640)	0.91
Solar	(13,557; 13,480)	0.89
Wind	(15,173; 15,158)	0.90

This table gives the performance of the RoBERTa-based task-specific support/oppose models trained in this study. This is after data augmentation for balancing. The Matthews correlation coefficient is a special case of the Pearson correlation coefficient and can therefore be interpreted the same way.

The volume of tweets is often dictated by the activity of select users, and while it represents the public discourse people can be subjected to, it may not fully capture the actual individual opinions. The positivity of discourse related to each energy technology is consequently derived for each individual user. This gives the number of users that *support*, *oppose*, or hold a *neutral* view toward a given energy over the last decade. Here, a neutral user is defined as a user that published between 40 and 60% of supporting tweets.

The effect of sudden perturbations on public discourse can also be considered. We assess the sensitivity of an energy to any given event using a half-life measurement. Two types of events are identified: short-term and long-term. Short-term is identified on a daily timeframe, while long-term is on a monthly scale. Tweets are aggregated by day and by month accordingly. We define the reference value as the 7-day average before the perturbation happens for the short-term and 6-month average for the long-term. This allows us to obtain a stable reference point. Daily data can be noisy, and events can overlap. To remove this effect, we stop the recovery at the first inflection point before fitting an exponential function of the form $y=a+be^{cx}$, where *y* is the tweets positivity fraction and *x* is the elapsed time. The time necessary to cover 50% of the dip between the reference value and the lowest positivity point is defined as the half-life. The mean and SD of the computed half-lives are given for the energy technologies, with the SD obtained using the square root of the average of the squared deviations from the mean.

A time walk is developed by aggregating the tweets relating to an energy and topic for each year and computing the prevalence of the climate change topic (number of tweets for an energy discussing climate change relative to all tweets for that energy), and the positivity of that topic (fraction of positive tweets in the climate change-related population). The list of keywords used to isolate climate change-related discussions among social media data is: “climate,” “warming,” “IPCC,” “CO_2_,” “greenhouse,” “acidification,” “carbon,” “emission,” and “methane.”

The public perception evolution, as it relates to discussed topics such as climate change, is computed using a time walk method that averages all relevant tweets over a yearly period and assesses their average positivity and the topic prevalence in comparison with the entire tweet yearly corpus of a given technology. A *K*-mean clustering analysis is performed to identify how the energy technologies cluster in relation to positivity and prevalence of discourse as it relates to climate topics. A silhouette score and ANOVA method are applied to statistically prove the clustering quality. Additionally, the average direction of this evolution is calculated using several “past” and “current” windows of 3, 4, and 5 years. The average positivity and prevalence of the first *j* years (i.e. 2009–2013 if *j* = 4) of the dataset are used in conjunction with that of the last *j* years (i.e. 2018–2022 if *j* = 4) to obtain the direction of the resulting vector. Multiple windows from 3 to 5 years are used to avoid potential yearly specificities and derive a more representative range with built-in uncertainties. The Hodges–Lehmann estimator is used to estimate the significance of the temporal evolution difference between climate and nonclimate tweets. It calculates the median of all possible pairwise differences between the two datasets and thus represents the central location of the difference between two samples. A bootstrap CI was also derived on this estimator.

## Supplementary Material

pgaf079_Supplementary_Data

## Data Availability

The trained machine learning models used in this study are available on Figshare at https://doi.org/10.6084/m9.figshare.28462421.v1 The code used throughout this study is available on GitHub at https://github.com/glher/Sentinel.

## Social Media Data

The data used in this work were publicly available through the Twitter API. No identifying information was used in the analysis.
