# Supplementary material for: Influence of climate change and accidents on perception differs among energy technologies
Source: PNAS Nexus. 2025 Mar 7;4(3):pgaf079. doi: 10.1093/pnasnexus/pgaf079 (PMC11928931; doi:10.1093/pnasnexus/pgaf079)
Supplement: pgaf079_Supplementary_Data [file pgaf079_supplementary_data.docx]

**Supplementary Information**

**Influence of climate change and accidents on perception differs among energy technologies**

GF L’Her^1^, NA Duncan^2^, HC Jenkins-Smith^3^, MR Deinert^1,4*^

^1^Nuclear Science and Engineering, The Colorado School of Mines

^2^Nuclear Science and Engineering Research Center, United States Military Academy

^3^Institute for Public Policy Research and Analysis, University of Oklahoma

^4^Payne Institute for Public Policy, The Colorado School of Mines

**Supplementary Note 1.** We categorize the perception of individual users based on their tweets into positive, negative, and neutral. We consider a user to be in support of an energy if they posted more than 60% of positive views, in opposition if they posted less than 40% supporting messages, and neutral otherwise. In other words, consider that a given user posted five tweets in 2010 containing the relevant keywords for a technology (Supplementary Note 6). Four of these tweets were classified as positive (1), and the last one was seen as negative (0). The user score is thus 80% positive, and they are considered to be a positive user for this energy.

Table S1 shows that over the entire period of analysis (January 2009 – July 2022), a significant majority (68%) of users that tweeted about coal did so negatively. Individuals discussing natural gas and nuclear energy are polarized, with respectively 50.0% and 44.5% of positive users. Wind and Solar energy show a strong individual support (72.6% and 75% respectively), while hydroelectric power sits a little below at 61.3% of the account holders tweeting supportively. Table S1 aggregates user data over the entire period of analysis and thus does not account for users potentially changing their mind over time. It is informative to look at each year separately, e.g., Table S2 shows the individual opinions in 2010 and Table S3 shows them in 2020. These two tables notably exhibit a fall in sentiment for hydroelectric power (a loss of 20 percentage points) and show a more circumspect discourse on new renewables over time. The data also show the rebound of the nuclear industry after the Fukushima accident, with a similar share of Twitter users shining a positive light on nuclear energy in 2020 compared to 2010. The significant impact on renewable energies may arise from a possible change in demographics and behaviors of Twitter users from its infancy (pre-2010) to current days. In 2020, 57.6% of users engaged in the discussions showed support for nuclear energy, a higher number than natural gas at 51.1%. It is also interesting to note that of the 27,352 users who posted about both nuclear and natural gas in 2020, 33.1% presented positive messages for both, 18.8% supported nuclear but posted negative tweets about natural gas, 11.3% opposed nuclear but showed natural gas in a positive light, and 14.2% opposed both, the rest being neutral for at least one of these technologies.

| Energy | Number of users | Negative | Neutral | Positive |
| --- | --- | --- | --- | --- |
| Coal | 451,035 | 67.97% | 8.28% | 23.75% |
| Natural gas | 882,683 | 39.90% | 10.11% | 49.99% |
| Nuclear | 1,226,834 | 46.51% | 8.99% | 44.50% |
| Hydroelectric | 269,891 | 32.46% | 6.26% | 61.28% |
| Solar | 1,041,355 | 20.65% | 4.35% | 75.00% |
| Wind | 897,693 | 21.30% | 6.09% | 72.61% |

**Table S1. Positivity of individual users January 1, 2009 – July 31, 2022.** This table looks at all energy-related tweets over the analyzed period and assesses the global positivity toward the energy discussed by each user. A total of 4,048,677 users published a tweet on at least one of the considered energies over the period of interest.

| Energy | Number of users | Negative | Neutral | Positive |
| --- | --- | --- | --- | --- |
| Coal | 26,522 | 72.06% | 5.79% | 22.15% |
| Natural Gas | 49,900 | 40.08% | 8.59% | 51.33% |
| Nuclear | 59,282 | 34.76% | 8.14% | 57.09% |
| Hydroelectric | 11,907 | 23.05% | 5.02% | 71.92% |
| Solar | 56,850 | 12.87% | 2.66% | 84.47% |
| Wind | 74,650 | 14.02% | 4.58% | 81.39% |

**Table S2. Positivity of individual users in 2010.** This table looks at all energy-related tweets over the year 2010 and assesses the global positivity toward the energy discussed by each user. A total of 178,279 users published a tweet on at least one of the considered energies over the period of interest.

| Energy | Number of users | Negative | Neutral | Positive |
| --- | --- | --- | --- | --- |
| Coal | 67,866 | 73.56% | 5.21% | 21.23% |
| Natural Gas | 134,787 | 41.61% | 7.28% | 51.11% |
| Nuclear | 182,216 | 36.99% | 5.38% | 57.63% |
| Hydroelectric | 45,165 | 45.72% | 4.40% | 49.88% |
| Solar | 122,136 | 30.52% | 3.03% | 66.45% |
| Wind | 109,599 | 27.79% | 3.99% | 68.22% |

**Table S3. Positivity of individual users in 2020.** This table looks at all energy-related tweets over the year 2020 and assesses the global positivity toward the energy discussed by each user. A total of 494,005 users published a tweet on at least one of the considered energies over the period of interest.

**Supplementary Note 2.** We look at multiple specific events related to given energy technologies to assess their resilience in the eye of the public discourse. External events that could have had an impact on the discussion are selected and we examine their effect, if any, on the daily conversations. To ensure a systematic identification of external events, we comb the first 15 pages of results from Google News related to relevant keywords: ‘coal plant explosion’, ‘coal accident’ for coal, ‘natural gas plant accident’ for natural gas, ‘nuclear plant accident’ for nuclear, and ‘hydroelectric accident’ for hydroelectricity. This method did not return relevant results for solar and wind energies. Figure S1 to S7 show a small, selected sample of identified events for each energy.

Figure S1 shows the impact of various smaller (short-term) events that affected nuclear power plants. The false alarm that was sent by error to residents of the Province of Ontario in Canada presents the lowest half-life among the selected events in Fig. S1, at 0.53 day. This event still registered significantly on the online discourse even though the all-clear was sent out 108 minutes later. Some events may not register, even for nuclear-related news. For example, the Hanford Nuclear Site tunnel collapse did not cause any adverse reaction on Twitter. Similarly, a uranium convoy attacked by an armed militia in Brazil did not cause any discernable dip, and a Tritium leak detected at the Perry Nuclear Power Plant in January 2014 did not raise any visible alarm on the nuclear discourse. These events can hint at potential ways for the nuclear industry to get ahead of the narrative by improving communication.


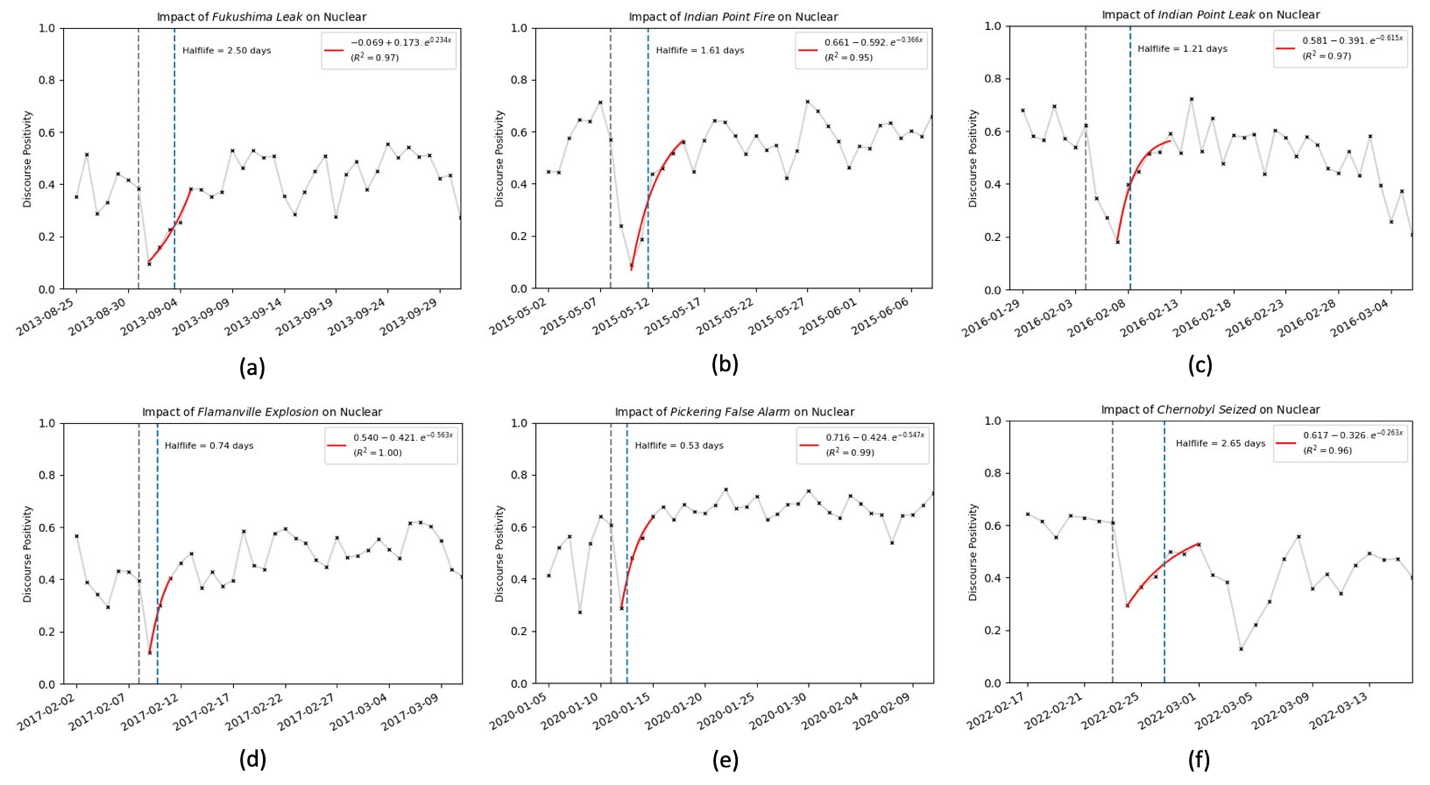


**Figure S1. Half-life of nuclear-related events impacts.** (a) Radioactive leak at Fukushima Daiichi Nuclear Power Plant^1^, (b) Fire at Indian Point Nuclear Power Plant^2^, (c) Leak at Indian Point Nuclear Power Plant^3^, (d) Explosion at Flamanville Nuclear Power Plant^4^, (e) False Cellphone Alarm about Pickering Nuclear Power Plant^5^, (f) Chernobyl is seized by Russian forces in Ukraine^6^. The grey dashed lines represent the day prior to the event for reference, and the blue dashed line shows the half-life when an effect is seen.

Fossil fuels are a lot less sensitive to much worse (deadly) events. In this study, we identified several significant events for both coal and natural gas, most of them resulting in casualties. Despite this, very few events recorded any impact on the public discourse. Figure S2 shows the impact of accident (most of them mining accidents) on online discourse relative to coal energy excluding the keyword ‘coal mine’ and ‘coal mining’. It shows only one event exhibiting an effect with a half-life of 1.9 days, the West Virginia mine accident in 2010 that took 29 lives. When also accounting for coal mine topics, the events are picked up more consistently in the discourse, Fig. S3. This does show that even very deadly coal mine accidents are not readily linked to coal power plants. Natural Gas exhibit similar sensitivity to external events with a few accidents making a dent on the average trend, Fig. S4.


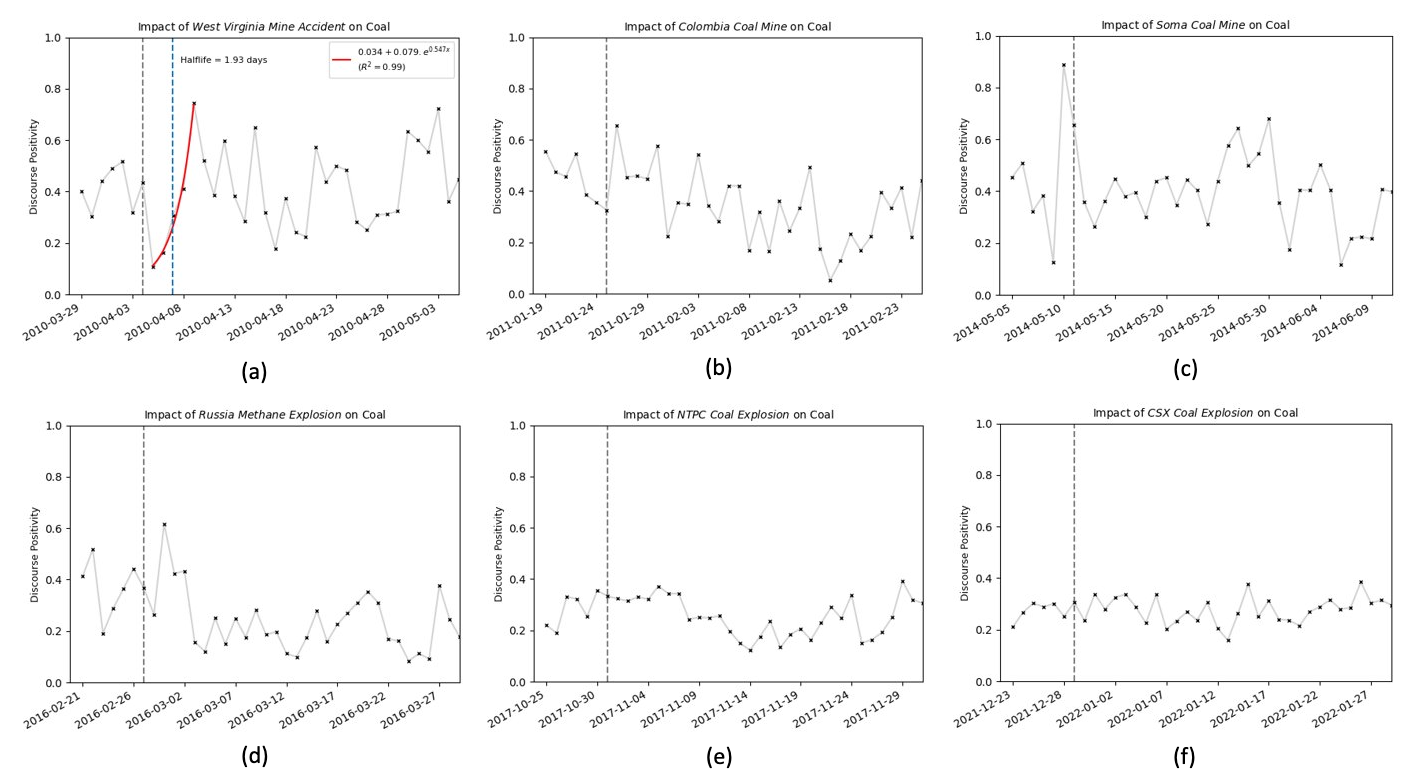


**Figure S2. Half-life of coal-related events impacts [excl. ‘coal mine’ tweets].** (a) Collapse of a coal mine in West Virginia (29 deaths)^7^, (b) Collapse of a coal mine in Colombia (20 deaths)^8^, (c) Collapse of a coal mine in Turkey (301 deaths)^9^, (d) Methane explosions in Russia (36 deaths)^10^, (e) Coal plant explosion in India (32 deaths)^11^, (f) Coal facility explosion in Maryland^12^. The grey dashed lines represent the day prior to the event for reference, and the blue dashed line shows the half-life when an effect is seen.


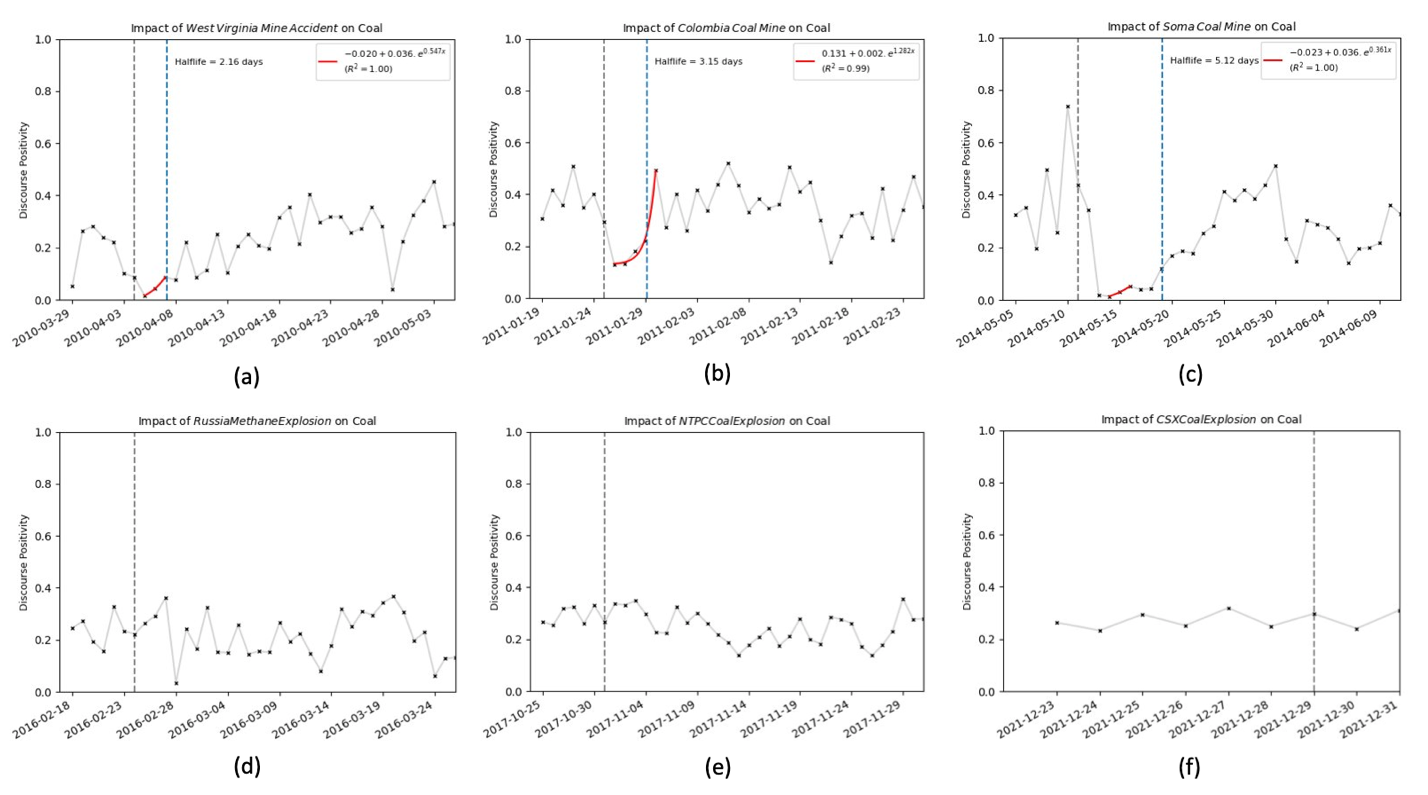


**Figure S3. Half-life of coal-related events impacts [incl. ‘coal mine’ tweets].** (a) Collapse of a coal mine in West Virginia (29 deaths)^7^, (b) Collapse of a coal mine in Colombia (20 deaths)^8^, (c) Collapse of a coal mine in Turkey (301 deaths)^9^, (d) Methane explosions in Russia (36 deaths)^10^, (e) Coal plant explosion in India (32 deaths)^11^, (f) Coal facility explosion in Maryland^12^. The grey dashed lines represent the day prior to the event for reference, and the blue dashed line shows the half-life when an effect is seen.


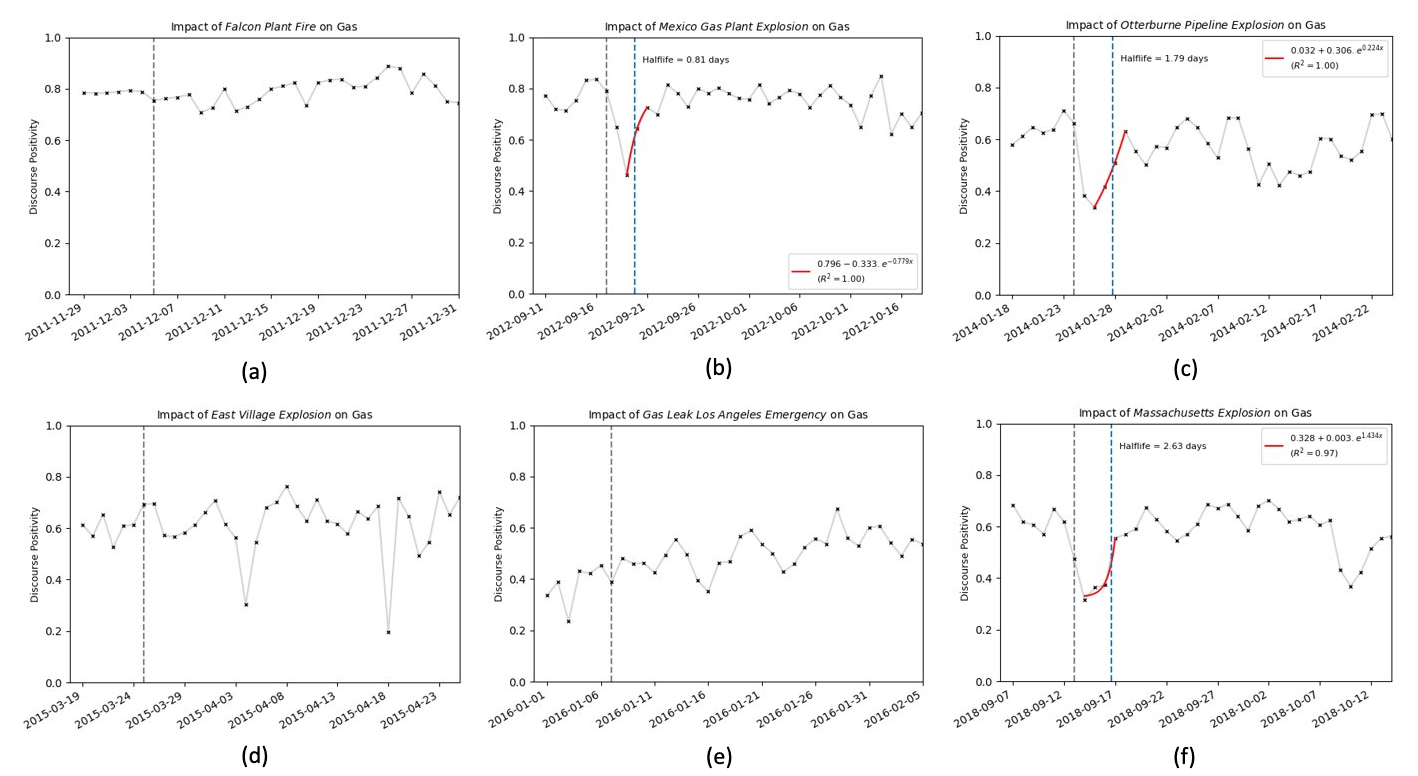


**Figure S4. Half-life of natural gas-related events impacts.** (a) Fire at a Gas Plant in Wyoming^13^, (b) Explosion at a Gas Plant in Mexico (31 deaths)^14^, (c) Explosion of a Gas Pipeline in the province of Manitoba, Canada^15^, (d) A Gas Explosion in New York City (2 deaths, March 26)^16^, a terrorist attack on a gas pipeline in Delta, Nigeria (April 4), and a gas explosion in Fresno, California (April 18), (e) Governor declares emergency over a Natural Gas leak near Los Angeles, California^17^, (f) Natural Gas explosions in Massachusetts (1 death, September 14)^18^, a petition against a gas plant goes viral in the UK (October 10). The grey dashed lines represent the day prior to the event for reference, and the blue dashed line shows the half-life when an effect is seen.

Hydroelectricity and related dam failures can be perceived as catastrophic by the public. The half-life of some of the events are shown in Figure S5. We show that hydropower is sensitive to events based on their actual severity, but that long-term half-lives are not seen.


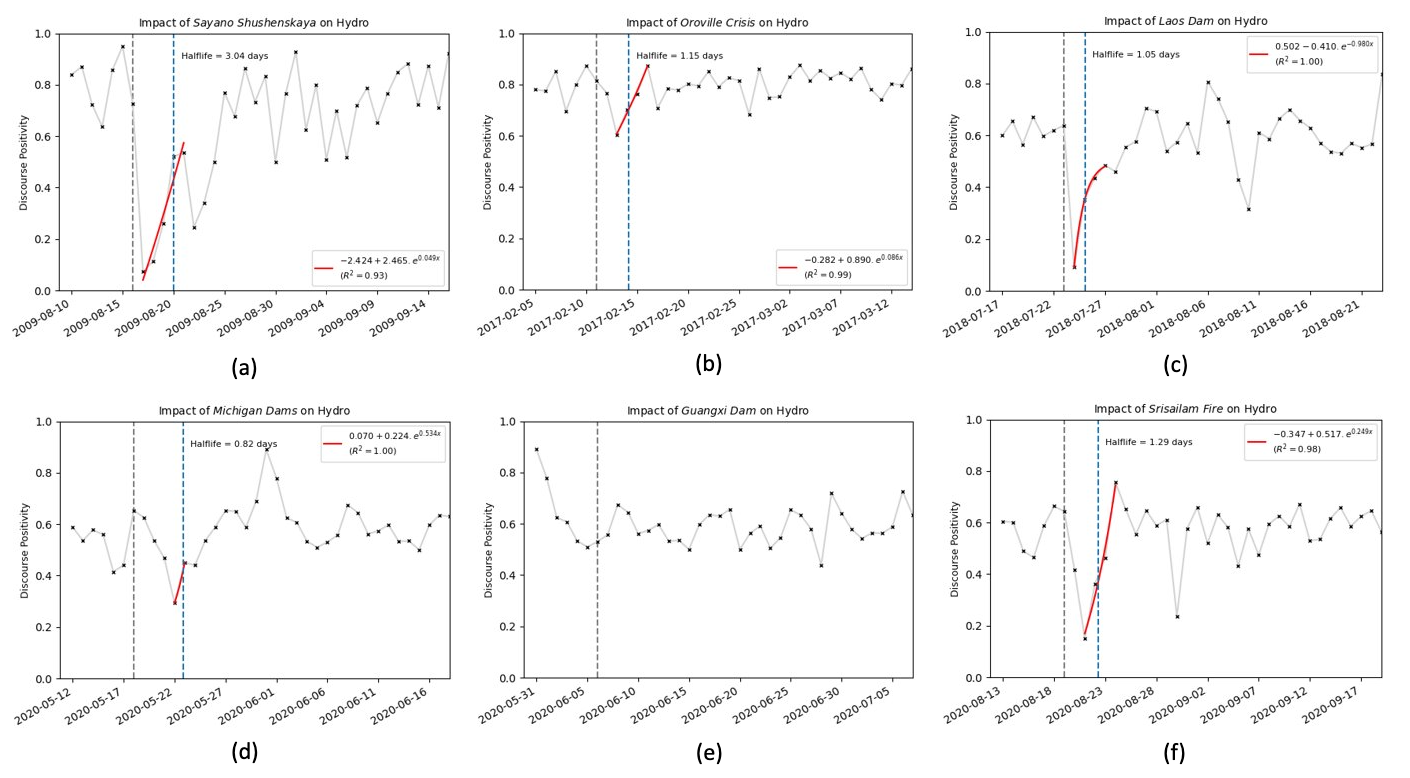


**Figure S5. Half-life of hydropower-related events impacts.** (a) Turbine failure in a Russian Hydropower Plant (75 deaths)^19^, (b) Oroville crisis in California, US (180,000 evacuations)^20^, (c) Dam failure in Laos in 2018 (71 deaths)^21^, (d) Collapse of a dam in Michigan, US^22^, (e) Collapse of a dam in the province of Guangxi in China^23^, (f) Fire at a Hydropower Plant (9 deaths, August 20)^24^, protest over the ecosystem impact of the Etalin hydropower project in India (August 30). The grey dashed lines represent the day prior to the event for reference, and the blue dashed line shows the half-life when an effect is seen.

Due to its distributed nature, wind energy is rarely exposed to catastrophic accidents, although workforce casualties happen. Here we observe the Twitter reaction to a few selected events, Fig. S6. Only one event was found to have a significant impact on wind energy discourse: the Texas 2021 winter storm. Wind farms, a significant share of electricity production in Texas, went down due to ice and were not able to help compensate the loss of other generators such as natural gas plants. A half-life of almost 3 days is observed for this event. Other (sometimes deadly) events involving the detachment of blades, fall, or turbine fires were not picked up on Twitter. We can note that on Fig. S6b, while the death of two maintenance workers stuck on top of a wind turbine after a fire erupted did not register, a significant dip is seen on November 23. We can explore the data and identify the initiating event for this sudden perturbation: On November 22, Duke Energy plead guilty to the killing of 14 golden eagles and 149 other birds at two wind farms in Wyoming.


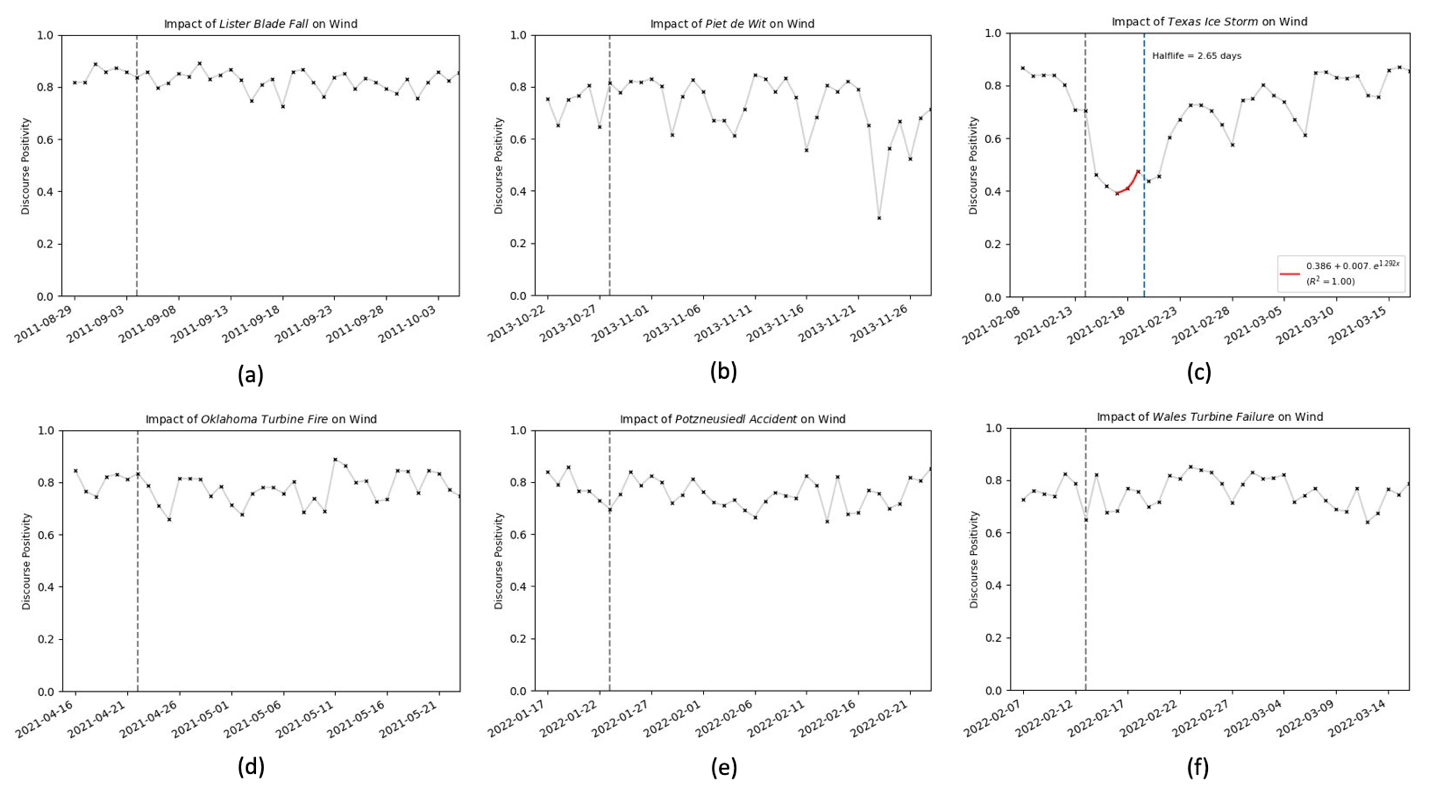


**Figure S6. Half-life of wind-related events impacts.** (a) A blade falls on a car in the UK^25^, (b) a turbine fire traps maintenance worker (2 deaths, October 28)^26^, Duke Energy pleads guilty to killing golden eagles (November 22), (c) a winter storm causes wind farms to shut down in Texas^27^, (d) a Turbine fire in Oklahoma^28^, (e) a worker falls to his death in Austria (1 death)^29^, (f) a wind turbine collapses in Wales^30^. The grey dashed lines represent the day prior to the event for reference, and the blue dashed line shows the half-life when an effect is seen.

Discourse positivity on solar energy is intuitively resilient to external events, Fig. S7. We show that while most events are indeed not detected on Twitter, in some rare occasion they can be. The fire at Ivanpah plant, the controversial largest concentrated solar plant in the world, is an example of this, with a half-life of around 2 days.


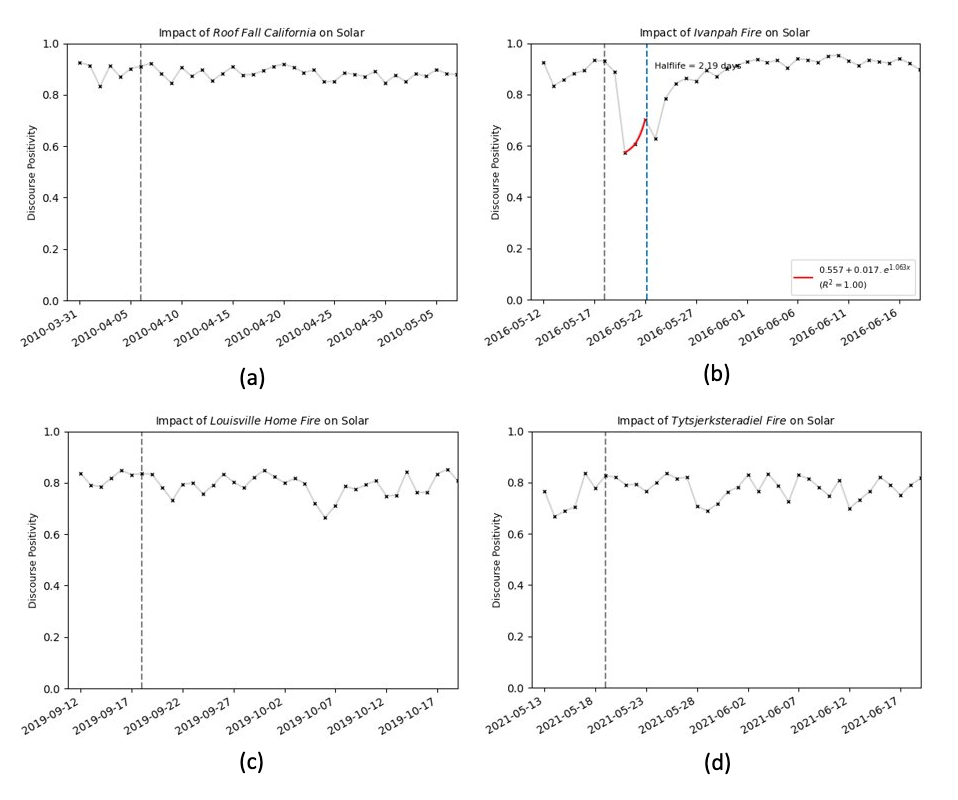


**Figure S7. Half-life of solar-related events impacts.** (a) A worker falls to his death in California (1 death)^31^, (b) Fire at Ivanpah solar farm in the US^32^, (c) House fire in Louisville, Colorado, US caused by solar rooftop^33^, (d) Fire at a solar panel manufacturer plant in the Netherlands^34^. The grey dashed lines represent the day prior to the event for reference, and the blue dashed line shows the half-life when an effect is seen.

**Supplementary note 3.** A K-means clustering analysis is carried on the positivity and prevalence paths. A silhouette score of 0.69 is obtained for the climate-related topics and 0.65 for the non-climate-related topics, showing a good clustering. An analysis of variance (ANOVA) test is performed on the x- and y-axis of the centroids to assess statistical difference of the clusters. P-values are computed and all sit below 1e-8, showing that the clusters are statistically significant.

Parts of the nuclear paths through prevalence and positivity of tweets not related to climate topics are parts of the most negative cluster, representing the direct impact of the Fukushima accident. This cluster analysis also shows the switch of hydroelectricity from the most positive cluster to the more neutral one.


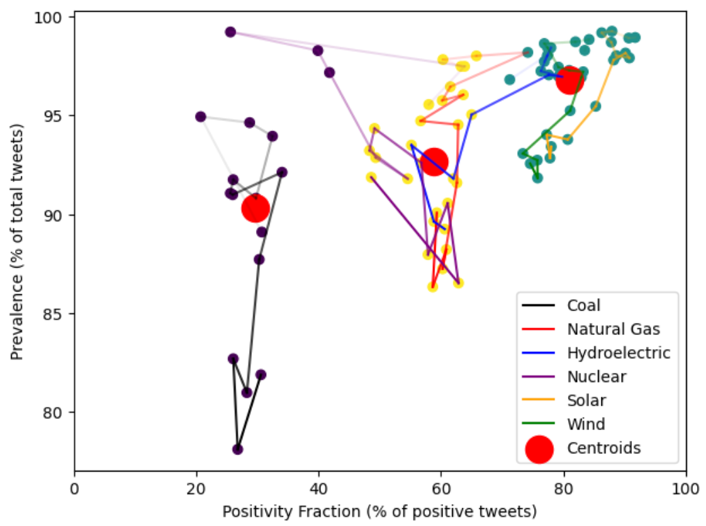

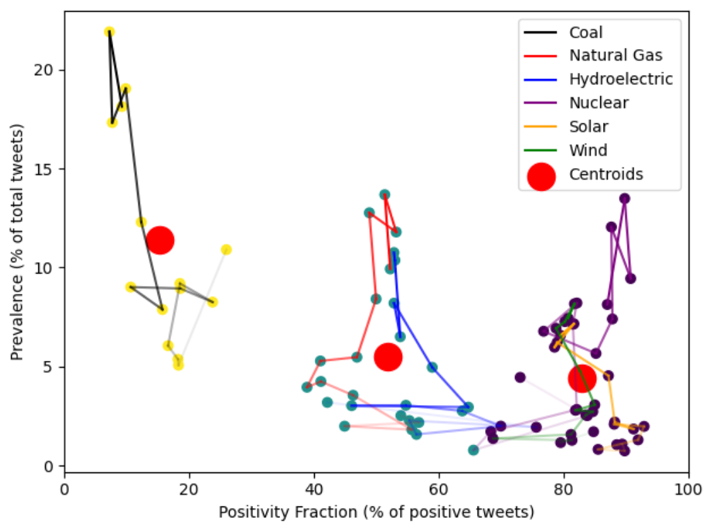


**Figure S8. K-means clustering (colored dots) of the energy technologies for prevalence and positivity evolution for climate-related discussions.** (left) Non-climate-related tweets, (right) climate-related tweets.

**Supplementary note 4.** The Hodges-Lehmann estimator is used to estimate the significance of the temporal evolution difference between climate and non-climate tweets. It calculates the central location of the difference between two samples, and thus represents the median of all possible pairwise differences between the two datasets. A bootstrap confidence interval was also derived on this estimator. Table S4 shows the Hedges-Lehmann estimator and corresponding confidence intervals for each technology. A negative estimator indicates that non-climate related tweets are more positive than climate-related tweets. This behavior is shown for Coal. The higher the absolute value of the estimator, the more marked the difference between the two datasets is. Consequently, even though nuclear is the only energy technology presenting a significant positive change over time, hydroelectricity and coal are more impacted by the climate discourse.

| Energy Technology | Hodges-Lehmann [C.I.] |
| --- | --- |
| Coal | -11.334 [-12.688 — -10.173] |
| Natural Gas | 4.273 [3.105 — 5.709] |
| Hydroelectricity | 11.596 [11.125 — 12.512] |
| Nuclear | 4.493 [3.260 — 6.683] |
| Solar | 2.076 [0.442 — 3.794] |
| Wind | 6.849 [5.763 — 9.558 |

**Table S4. Hodges-Lehmann estimator and corresponding confidence interval (C.I.) for the temporal evolutions of the positivity between climate and non-climate related tweets.** The values represent percentage points change in positivity messaging (climate evolution subtracted from non-climate evolution).

**Supplementary Note 5.** The sudden drops in sentiment levels shown with all technologies in Fig. 1 often correspond to specific events, and a subset of these are given in Supplementary Note 2. These, coupled with other sources of variation - like usage - contribute to the overall scatter in the respective signals and this can be measured with a standard deviation for the public opinion in the daily-aggregated data. Figure S9 shows the evolution of the variability over time, at a yearly scale. We show that the variability of online opinions related to solar and wind stayed relatively constant over time. However, coal, nuclear, hydropower, and natural gas all exhibit a decreasing trend in their standard deviation toward the variability of ‘new renewables’. Nuclear power show significant jumps after the Fukushima Daiichi accident and the Russian takeover of Ukrainian nuclear facilities in 2022.


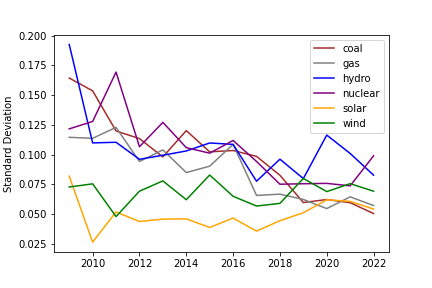


**Figure S9. Yearly evolution of the daily-aggregated tweets variability.** Tweets are aggregated by day, and the standard deviation of the daily positivity data observed in any given year plotted for each energy.

**Supplementary note 6.** The list of keywords used to fetch all relevant tweets in the 2009-2022 time period is given in Table S5.

| Technology | Keywords |
| --- | --- |
| Coal | coal power, coal plant, coal energy, coal-fired power, coal-fired plant, coal-fired energy, coal mine, coal mining |
| Natural Gas | gas pipeline, natural gas, gas power, gas-fired power, gas plant, gas-fired plant |
| Hydroelectricity | hydroelectric, hydropower, hydro power, hydro energy, hydro electric, hydro-electric |
| Nuclear | nuclear energy, nuclear plant, nuclear power, nuclear reactor, atomic power, thorium |
| Wind | wind energy, wind farm, wind turbine, wind power, onshore wind, offshore wind |
| Solar | solar energy, solar plant, solar panel, solar roof, photovoltaic |

**Table S5. Keywords list.** This table lists the keywords that were used to identify and download tweets related to each energy. The queries were run for any tweets posted between January 2009 and July 2022

**Supplementary note 7.** The tweets are classified depending on the positive light shone on the specific energy discussed, based on the question: *“Is the tweet shining a positive light, factual or subjective, on the considered energy?”*. A few examples are shown for Coal (Table S6), Natural Gas (Table S7), Nuclear (Table S8), Hydroelectric (Table S9), Solar (Table S10), and Wind (Table S11).

| Coal | Support | Thar coal power plant to become operational by June 2019 |
| --- | --- | --- |
|  |  | another wave of coal plant closures is finding the way, how can someone stop it? |
|  | Oppose | Environmental concerns raised by locals over dust levels at Cork coal plant |
|  |  | Eskom coal power causing estimated 2 200 premature deaths per year - Greenpeace |

**Table S6. Sample of classified tweets for Coal.**

| Natural Gas | Support | "I look forward to the day when domestic natural gas displaces foreign oil in the United States."" -John Deutch, MIT #CSIS |
| --- | --- | --- |
|  |  | Do you smell that fresh mountain air? Colorado sure does! As EID's latest analysis shows, #naturalgas is helping lower emissions in the state. |
|  | Oppose | can, but will he? Central VA has been left to try & fend off Dominion's Gas Pipeline without his support. |
|  |  | Fracked natural gas is not "clean energy." Methane is a huge threat. #NoDAPL |

**Table S7. Sample of classified tweets for Natural Gas.**

| Nuclear | Support | #Nuclear to help #Japan meet #climate goals |
| --- | --- | --- |
|  |  | China's first domestically made nuclear reactor goes online |
|  | Oppose | Senate says no to nuclear power damn right |
|  |  | Another one bites the dust: #nuclear plant on Lake Michigan to shut down #TooObsoleteToMeter |

**Table S8. Sample of classified tweets for Nuclear.**

| Hydroelectric | Support | Harnessing #hydropower. So awesome to see units installed in this beautiful setting! |
| --- | --- | --- |
|  |  | Solar not your thing? Camped or ped by flowing water? Micro hydro power to the rescue! |
|  | Oppose | Amazonians Prepare for #War to Massive Hydroelectric Dam on Xingu River #PERU #BRASIL #Indigenous #green |
|  |  | Boom or bust? more on the effects of increased #hydropower on #biodiversity #renewables |

**Table S9. Sample of classified tweets for Hydroelectric.**

| Solar | Support | the future of solar energy! #solar #renewable #sustainability #MENA |
| --- | --- | --- |
|  |  | Solar Panel Roads are coming - We could produce 3 times more power than we use as a nation. #gosolar |
|  | Oppose | Solar City went out of business because non-solar residents didn't want to subsidize their solar panel scam! |
|  |  | what did she really just say solar pv? yes let's bankrupt GA with an obama. vote |

**Table S10. Sample of classified tweets for Solar.**

| Wind | Support | Wind power at its all-time lowest cost? It's as if there are no more reasons not to use it! |
| --- | --- | --- |
|  |  | #WindPower Smashes Records Worldwide #renewable #energy #renewables #RenewableEnergy #wind #windenergy |
|  | Oppose | scotland risked being screwed by wind power. |
|  |  | oil billionaire koch brothers determined to kill wind power |

**Table S11. Sample of classified tweets for Wind.**

**Supplementary note 8.** Transformers-based models, and notably RoBERTa which was trained on an even larger corpus than the original BERT, are currently the state-of-the-art in Natural Language Processing^35^. Figure S10 shows a simplified architecture, based on representation from the original BERT paper^36^. A Pre-Trained RoBERTa language representation model is fine-tuned using task-specific training data. The weights in the encoders are updated accordingly during the additional training to generate a well-suited model after knowledge transfer.


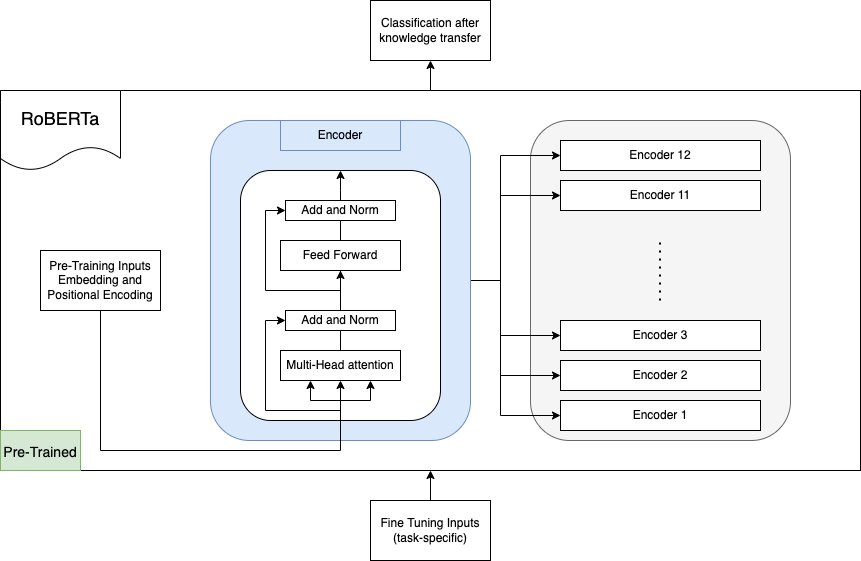


**Figure S10. Simplified representation of Fine-Tuning with the RoBERTa Transformer.**

**References**

1. Neuman, S. Radioactive Water Leak At Fukushima Worse Than First Thought. *NPR* (2013).

2. Al Jazeera Staff. Explosion rocks New York nuclear power plant. *Al Jazeera* (2015).

3. Shortell, D. Indian Point nuclear plant leak causes radioactivity in groundwater. *CNN* (2016).

4. Chappell, B. Explosion Hits Nuclear Power Station In Northwest France. *NPR* (2017).

5. Austen, I. Ontario Alert Warned of a Nuclear ‘Emergency,’ Then Backed Down. (2020).

6. IAEA. Update 1: IAEA director general statement on the situation in Ukraine. *IAEA.org* https://www.iaea.org/newscenter/pressreleases/update-iaea-director-general-statement-on-situation-in-ukraine-25-feb-2022 (2022).

7. Urbina, I. Toll Mounts in West Virginia Coal Mine Explosion. *New York Times* (2010).

8. Kimball, J. Colombia coal mine blast kills 20, regulator says. *Reuters* (2011).

9. Neuman, S. & Chappell, B. Death Toll Nears 285 In Turkish Coal Mine Explosion. *NPR* (2014).

10. Chappell, B. 36 People Killed In Coal Mine Explosions And Fire, Russia Says. *NPR* (2016).

11. BBC Staff. India power plant explosion leaves at least 29 dead. *BBC* (2017).

12. WUSA9 Staff. Explosion at CSX coal terminal rattles Baltimore buildings. *WUSA9* (2021).

13. Fugleberg, J. Two injured in fire, explosions at Wyoming natural gas site. *Casper Star-Tribune* (2011).

14. BBC Staff. Mexico probes Pemex gas plant explosion which killed 26. *BBC* (2012).

15. Nickel, R. TransCanada gas pipeline explodes in Manitoba, none hurt. *Reuters* (2014).

16. Sanchez, R. Injuries as buildings collapse after blast at New York’s East Village. *CNN* (2015).

17. Lovett, I. Governor Declares Emergency Over Los Angeles Gas Leak. *New York Times* (2016).

18. Seelye, K. & Stockman, F. After Massachusetts Gas Explosions, Weary Residents Ask, What Happened? (2018).

19. Parfitt, T. Eight dead, 54 missing, as turbine hall caves in at Russian hydropower station. *The Guardian* (2009).

20. Schmidt, S., Hawkins, D. & Phillips, K. 188,000 evacuated as California’s massive Oroville Dam threatens catastrophic floods. *Wahsington Post* (2017).

21. BBC Staff. Laos dam collapse: Many feared dead as floods hit villages. *BBC* (2018).

22. Samenow, J. Central Michigan’s dam and flood disaster as seen from space. *Washington Post* (2020).

23. Woo, R. Dam collapse in China could point to a ‘black swan’ disaster. *Reuters* (2020).

24. BBC Staff. India power plant fire: Nine reported dead in major blaze in Telangana. *BBC* (2020).

25. Young, R. Six-foot blade flies off new Lister turbine. *The Comet* (2011).

26. NL Times Staff. Two dead in fire in wind turbine Ooltgensplaat. (2013).

27. Mulder, B. Frozen wind turbines hamper Texas power output, state’s electric grid operator says. *Austin American-Statesman* (2021).

28. Raache, H. Wind turbine catches fire in Johnston County. *KFOR* (2021).

29. Richard, C. Probe launched after death at Austrian wind farm. *Wind Power Monthly* (2022).

30. BBC Staff. Giant wind turbine collapse to be investigated. *BBC* (2022).

31. CDC Staff. A Solar Panel Installer Dies When He Falls Off a Roof. *Center for Disease Control* (2010).

32. Mejia, B. Fire breaks out at world’s largest solar power plant near Nevada border. *Los Angeles Times* (2016).

33. Mauro, M. Solar panels catch fire on Louisville home. *KDVR* (2019).

34. Bellini, E. Major fire at solar-powered warehouse in the Netherlands raises concerns among nearby residents. *PV Magazine* (2021).

35. Wolf, T. *et al.* HuggingFace’s Transformers: State-of-the-art Natural Language Processing. Preprint at http://arxiv.org/abs/1910.03771 (2020).

36. Vaswani, A. *et al.* Attention is all you need. *Adv. Neural Inf. Process. Syst.* **30**, (2017).
